# Treatment response to Janus kinase inhibitor in a child affected by Aicardi‐Goutières syndrome

**DOI:** 10.1002/ccr3.7724

**Published:** 2023-07-31

**Authors:** Jessica Galli, Marco Cattalini, Erika Loi, Rosalba Monica Ferraro, Silvia Giliani, Simona Orcesi, Lorenzo Pinelli, Raffaele Badolato, Elisa Fazzi

**Affiliations:** ^1^ Department of Clinical and Experimental Sciences University of Brescia Brescia Italy; ^2^ Unit of Child Neurology and Psychiatry ASST Spedali Civili of Brescia Brescia Italy; ^3^ Pediatrics Clinic ASST Spedali Civili of Brescia Brescia Italy; ^4^ Department of Molecular and Translational Medicine University of Brescia Brescia Italy; ^5^ “Angelo Nocivelli” Institute for Molecular Medicine, ASST Spedali Civili of Brescia Brescia Italy; ^6^ Child Neurology and Psychiatry Unit IRCCS Mondino Foundation Pavia Italy; ^7^ Department of Brain and Behavioral Sciences University of Pavia Pavia Italy; ^8^ Neuroradiology Unit, Section of Pediatric Neuroradiology ASST Spedali Civili of Brescia Brescia Italy

**Keywords:** Aicardi‐Goutières syndrome, baricitinib, JAK‐inhibitors, neurodevelopmental outcome

## Abstract

**Key Clinical Message:**

Baricitinib, a Janus kinase inhibitor (JAK‐inhibitor), seems to contribute to an improvement of a child affected by Aicardi‐Goutières syndrome (AGS), reducing the interferon score and determining a recovery of cognitive, communicative, and relational dysfunctions, while the gross motor deficit persisted.

**Abstract:**

We report the treatment response to baricitinib, a JAK‐inhibitor, in a 4‐year‐old girl affected by Aicardi‐Goutières syndrome (AGS2, *RNASEH2B* mutation). Using quantitative measures, we detected a significant amelioration characterized by a complete recovery of cognitive, communicative, and relational skills after 8 and 16 months from the beginning of therapy.

## 1 INTRODUCTION

Aicardi‐Goutières syndrome (AGS) is a rare genetic encephalopathy, characterized by a wide range of neurological and extra‐neurological manifestations.^1^, ^2^ Particularly, five relatively stereotyped clinical scenario due to mutations in the AGS‐related genes have been reported: (1) “classic” AGS with prenatal or infantile onset presenting with irritability, feeding difficulties, microcephaly, abnormal movements, and epilepsy, as well as hematological and liver disturbances; (2) “late onset” AGS with subacute or slowly progressive neurological regression evolving to spastic‐dystonic syndrome; (3) dystonia due to bilateral striatal necrosis; (4) “non‐syndromic” spastic paraparesis; (5) intracerebral large vessel disease.^1^ To date, nine genes encoding for proteins involved in nucleic acids metabolism and/or sensing have been associated with AGS: *TREX1*, (AGS1), *RNASEH2A* (AGS4), *RNASEH2B* (AGS2), *RNASEH2C* (AGS3), *SAMHD1* (AGS5), *ADAR1* (AGS6), *IFIH1* (AGS7), *LSM11* (AGS8) and *RNU7*‐*1* (AGS9).^3^, ^4^ Mutations in these genes converge to a common pathway characterized by an abnormal over production of Type I interferon (IFN), involved in AGS pathogenesis.^2^ Thus, new therapeutic anti‐interferon strategies have been proposed. The most promising treatment for improving symptoms in Type I interferonopathies seems Janus kinase (JAK) inhibitors that block JAKs, molecules that induce the transcription of IFN‐stimulated genes.^5^, ^6^ However, data on AGS are limited to few case reports^2^, ^4^, ^7^, ^8^, ^9^, ^10^ and a single clinical trial.^11^ All the case reports, except one, documented a neurological improvement with ruxolitinib through quantitative measures; conversely, in the clinical trial patients were treated with baricitinib but the clinical outcomes were extracted from diaries completed by parents. Herein, we report the clinical picture and treatment response to baricitinib in one child affected by AGS, using standardized measures for neuropsychiatric involvement.

## 2 CASE REPORT

She is a 4‐year‐old girl, born to Moroccan healthy unrelated parents. Pregnancy, delivery, and perinatal period were uneventful. She appropriately acquired the early developmental milestones. Neither feeding or sleeping disorders nor recurrent fevers were reported.

At 16 months of age, she presented with gait disturbances and left pyramidal signs: hypertonia, weakness, hyperreflexia, and positive Babinski. The Gross Motor Function Measure (GMFM)‐88 revealed a mild impairment and the Griffiths Mental Development Scales‐III normal/borderline scores (Table 1). After 1 week, she manifested extreme irritability and a neuropsychomotor regression evolving in few months to spastic tetraplegia, severe developmental delay, and loss of words production. Metabolic analyses (lactic acid, plasmatic and urinary amino acids, urinary organic acids, and acyl carnitine), ocular examination, and instrumental screening (echocardiogram, abdominal, thyroid and pelvis ultrasound) were negative. Brain MRI showed signal abnormalities in the brainstem and in the supratentorial white matter, hyperintense on T2‐weighted images, with no diffusion restriction nor contrast enhancement after contrast medium administration; spinal cord MRI was normal. On suspecting AGS, IFN signature was performed,^12^ which resulted in an increase in the score (24.1). Genetic analysis confirmed the diagnosis of AGS type 2, revealing homozygous mutation on *RNASEH2B*:NM_024570.3:c.[529G>A, 529G>A]:p.[A177T]; [A177T]. At 20 months, she underwent a follow‐up brain MRI documenting reduced signal abnormality in the above reported areas; brain CT scan did not show any calcification. At 22 months, she started infection prophylaxis with intravenous immunoglobulins (IVIG) (dosage: 400 mg/kg/4 weeks). However, the drugs were suspended by family's choice due to SARS‐CoV‐2 emergency. At 28 months, we documented a slight neurological improvement: disappearance of irritability and acquisition of the ability to pronounce of three to four words and to grasp an object. At 37 months, treatment with IVIG and baricitinib (dosage: 0.7 mg/kg divided in three daily doses) was started. The use of baricitinib was approved by the local authority on Rare Diseases (“Centro Regionale per le Malattie Rare M. Negri”). Written informed consent for genetic testing, participation and publication of the study was obtained from parents. The child underwent neurological examination, GMFM‐88 and AGS severity scale^13^ before (36 months) and after 1 (38 months), 2, 8 and 16 months from treatment. Griffiths‐III and Vineland Adaptive Behavior Scales‐II (VABS‐II) were administered before baricitinib and after 8 and 16 months. IFN signature was also analyzed.

**TABLE 1:** Standardized assessment performed before and during the treatment.

Standardized assessment	Age (months)							
	Before therapy				During therapy			
	16	22	28	36	38	39	45	53
GMFM‐88 domain, %								
Lying & rolling	92	47	47	55	55	57	57	60
Sitting	70	25	25	30	35	35	35	38
Crawling & kneeling	69	0	0	5	5	5	5	5
Standing	44	0	0	5	5	10	10	10
Walking, running & jumping	21	0	0	1	1	1	1	1
Total score	59	15	15	19	20	22	22	23
Griffiths‐III, standard score								
Foundation of learning	74	<50	<50	74	–	–	102	105
Language and communication	91	<50	<50	57	–	–	94	109
Hand‐eye coordination	79	<50	<50	<50	–	–	61	64
Personal‐social‐emotional	102	<50	<50	66	–	–	90	104
Gross‐motor	95	<50	<50	<50	–	–	<50	<50
Total score	87	<50	<50	<50	–	–	55	61
AGS severity scale	–	5	6	6	7	9	9	9
VABS‐II, *p*								
Communication skills	–	–	–	4	–	–	6	68
Daily living skills	–	–	–	1	–	–	2	70
Socialization	–	–	–	14	–	–	68	93
Motor skills	–	–	–	0.5	–	–	<0.1	<0.1
Total score	–	–	–	16	–	–	3	39

Abbreviations: AGS, Aicardì‐Goutieres syndrome; GMFM‐88, Gross Motor Function Measure‐88; Griffiths‐III, Griffiths Mental Development Scales‐III; VABS‐II, Vineland Adaptive Behavior Scale‐II.

Before treatment, we revealed a mild improvement of learning, communication, and personal skills while the fine and gross motor deficit remained almost stable. She completed 3‐hole board game, used about 10 words and participated to some self‐care tasks (Table 1).

After administering baricitinib, we documented a progressive and considerable amelioration of the neurological picture. At Griffiths‐III the girl obtained normal scores in foundation of learning, language and communication, personal‐social‐emotional subdomains, borderline score in hand‐eye coordination and delayed score in gross motor subdomain. Similar findings were found at VABS‐II: she reached normal/high scores in communication, socialization and daily living skills while the low score in motor skills persisted at every time points. The child was able to perform a 11‐hole board game, to organize words into structured phrases (45 months), to eat without assistance, to control the sphincters (53 months), to play with other kids (53 months), and to draw lines/circles/human figure (45 months). Although the standardized tests did not reveal significant changes, the girl acquired the ability to maintain the sitting position without support for more than 10 s (38 months), to stand up with support (39 months), and to roll attaining the sitting position (53 months). The AGS severity scores increased from six to nine points (Table 1).

**FIGURE 1:**
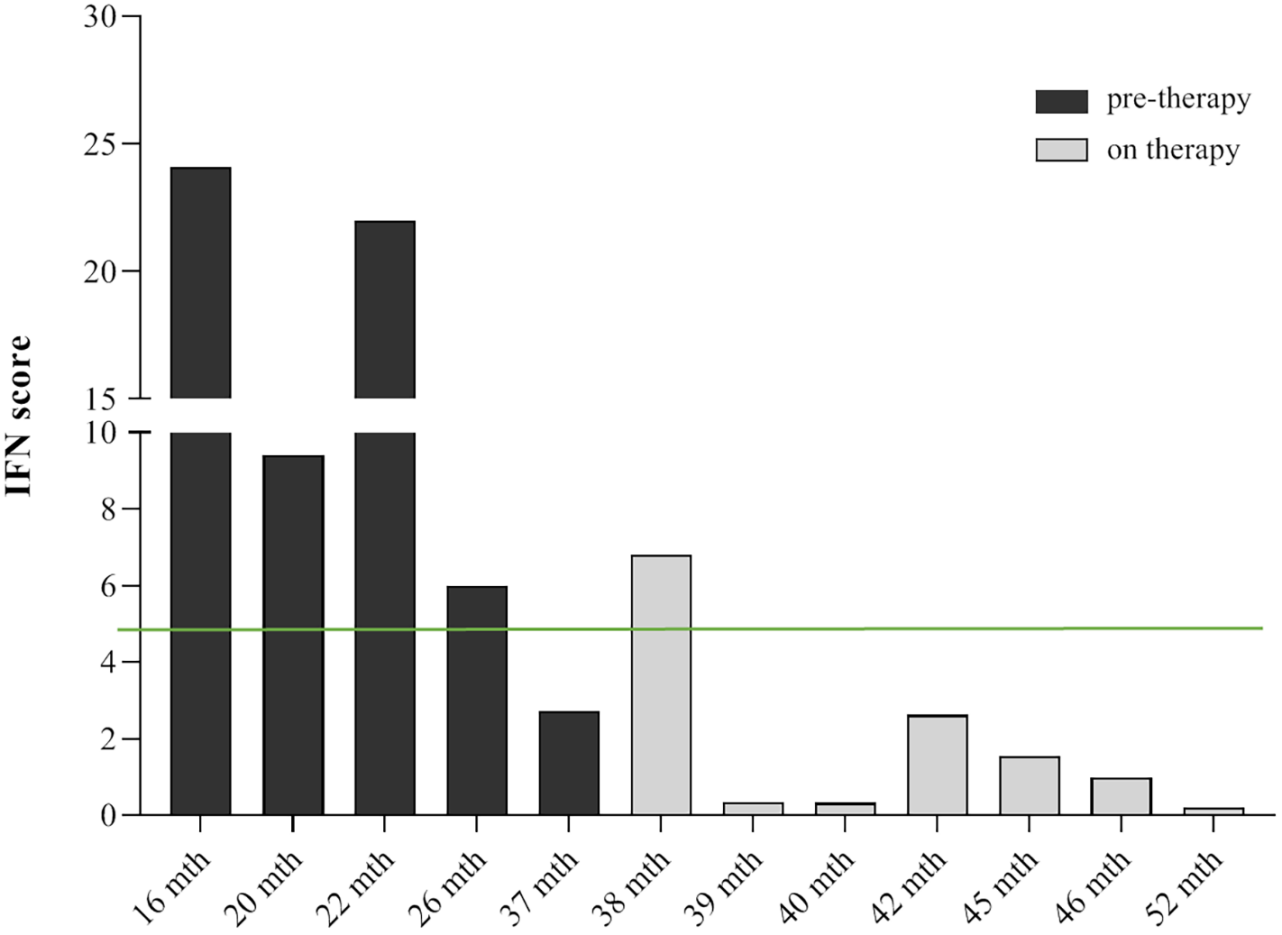
Trend of interferon‐signaling gene expression score before and during treatment with baricitinib. The interferon score was calculated as the median fold changes of expression of a panel of interferon stimulated genes (ISGs: IFI27, IFI44L, IFIT1, RSAD2, ISG15, and SIGLEC1). The green line defines the mean IFN score derived from a pool of 17 healthy controls plus two standard deviations above the mean was calculated. Scores higher than this value (4.67) are designated as positive.

After 3 months, the dosage of Baricitinb was reduced to 0.5 mg/kg divided in three daily doses due to asymptomatic thrombocytosis, increase of transaminases and creatine kinase, and after 9 months the IVIG injection was stopped due to collateral effects (headache, fever, nausea, vomiting 12–24 after the end of the infusion). The IFN score (Figure 1) showed a fluctuated trend before therapy, while during treatment it remained constantly to low levels (average: 1.8).

## 3 DISCUSSION

JAK‐inhibitors seem to be the most promising molecules against interferonopathies, reducing the inflammatory reaction related to uncontrolled IFN production.^6^ Our patient presented with a mild neurological and neuroradiological improvement after the first 6 months from the disease onset, as reported in some AGS subjects,^10^, ^14^ but a more significant amelioration occurred after 8 months from baricitinib. A complete recovery of cognitive, communicative, relational skills, and fine motor gains against the previous stagnation was observed. The gross motor deficit persisted over time, although a slow and extremely mild progression was noticed both before and during the treatment. The IFN score showed lower values compared to the first evaluations.

Baricitinib seemed to contribute to the child clinical improvement. Indeed, literature data reported that some AGS subjects may present a spontaneous recovery of neurologic dysfunctions especially within 6 months after the disease onset,^14^ while our patient showed the most improvement after about 2 years. Furthermore, the clinical features of our patient at last evaluation seemed to be milder compared to the typical profile of individuals with AGS related to *RNASEH2B*, characterized by gross and fine motor dysfunctions (such as rolling, bringing hands together, reaching an object) and language deficit (pronouncing less than six words).^14^ Conversely, our girl was able to roll, to stand up with support, to hold a pencil and draw, and to talk in sentences.

Baricitinib seemed to be well tolerated except for asymptomatic laboratory abnormalities managed with a dose‐reduction. Thrombocytosis has been previously reported, but it is not clear whether it is attributable to the effect of chronic inflammation or to JAK‐inhibitors.^11^, ^15^

Although this case confirmed that baricitinib could be beneficial in preventing progression of the disease, our findings should be considered carefully for several reasons: (1) Our study involves a single patient, (2) the child presented an “atypical” form of AGS characterized by a late and abrupt onset of neurologic decline in an otherwise healthy child without the typical history of the syndrome and of inflammation; moreover, brain calcifications were not observed.

Our results partially differ from those observed in a recent study on 11 subjects affected by AGS treated with JAK‐inhibitors, documenting a clear benefit on systemic disturbances and interferon level but no significant change in neurological status.^16^ Variation in disease expression, also between siblings, and differences in the AGS stage and genotype could explain these findings. Theoretically, as the involvement of JAKs could have slight differences, depending on AGS type, and the JAKs‐inhibitors have different affinity for JAKs, it could be that JAK‐inhibitor could have variable efficacy based on genotype/phenotype of the patient and drug used. In fact, treatment efficacy seems to be higher in subjects with less severe phenotype especially due to *SAMHD1*, *ADAR1*, and *IFIH1* mutations.^16^ However, to be strictly related to clinical practice the only two JAK‐inhibitor that have been used in AGS are baricitinib and ruxolitinib. Baricitinib seems to have a stronger inhibitory effect on cytokine release and a major capability to cross the blood–brain barrier than ruxolitinib (concentration in the cerebrospinal fluid: 10%–20% vs. 10% of that in blood).^17^ However, no studies are available on the comparison between the two drugs regarding their efficacy.

In conclusion, our evidence suggests that although limited to a single case, baricitinib may be useful to reduce the impact of AGS on neurological outcome. Due to the rarity of the disease, multicenter studies are required to understand the natural evolution of the syndrome and to prove efficacy and safety of JAK‐inhibitors.

## AUTHOR CONTRIBUTIONS

**Jessica Galli:** Conceptualization; data curation; investigation; methodology; validation; visualization; writing – original draft. **Marco Cattalini:** Conceptualization; data curation; investigation; methodology; validation; visualization; writing – original draft. **Erika Loi:** Writing – original draft. **Rosalba Monica Ferraro:** Investigation; methodology; writing – original draft. **Silvia Giliani:** Investigation; methodology. **Simona Orcesi:** Writing – review and editing. **Lorenzo Pinelli:** Investigation; writing – original draft. **Raffaele Badolato:** Conceptualization; supervision; writing – review and editing. **Elisa Fazzi:** Conceptualization; supervision; writing – review and editing.

## FUNDING INFORMATION

Authors declare no funding sources.

## CONFLICT OF INTEREST STATEMENT

The authors declare no competing interests.

## ETHICS STATEMENTS

The protocol for this research project (the use of baricitinib) has been approved by the local authority on Rare Diseases (“Centro Regionale per le Malattie Rare M. Negri” as per local regulation) and it conforms to the provisions of the Declaration of Helsinki.

## CONSENT

Written informed consent was obtained from parents to publish this report in accordance with the journal's patient consent policy.

## Data Availability

The raw data supporting the conclusions of this article will be made available by the authors, without undue reservation.
